# LSDV-Vectored SARS-CoV-2 S and N Vaccine Protects against Severe Clinical Disease in Hamsters

**DOI:** 10.3390/v15071409

**Published:** 2023-06-21

**Authors:** Warren R. J. de Moor, Anna-Lise Williamson, Georgia Schäfer, Nicola Douglass, Sophette Gers, Andrew D. Sutherland, Melissa J. Blumenthal, Emmanuel Margolin, Megan L. Shaw, Wolfgang Preiser, Rosamund Chapman

**Affiliations:** 1 Division of Medical Virology, Department of Pathology, Faculty of Health Sciences, University of Cape Town, Observatory, Cape Town 7925, South Africa; 2 Institute of Infectious Disease and Molecular Medicine, Faculty of Health Sciences, University of Cape Town, Cape Town 7925, South Africa; 3 International Centre for Genetic Engineering and Biotechnology, Observatory, Cape Town 7925, South Africa; 4 Wellcome Trust Centre for Infectious Disease Research in Africa, University of Cape Town, Cape Town 7925, South Africa; 5 Pathcare VetLab, Cape Town 7463, South Africa; 6 Division of Medical Virology, Faculty of Medicine and Health Sciences, Stellenbosch University Tygerberg Campus, Cape Town 7505, South Africa; 7 Biopharming Research Unit, Department of Molecular and Cell Biology, University of Cape Town, Cape Town 7701, South Africa; 8 Department of Medical Biosciences, Faculty of Natural Sciences, University of the Western Cape, Bellville, Cape Town 7535, South Africa

**Keywords:** SARS-CoV-2, LSDV, COVID-19, vaccine, challenge, nucleocapsid, spike, lumpy skin disease virus, poxvirus

## Abstract

The SARS-CoV-2 pandemic demonstrated the need for potent and broad-spectrum vaccines. This study reports the development and testing of a lumpy skin disease virus (LSDV)-vectored vaccine against SARS-CoV-2, utilizing stabilized spike and conserved nucleocapsid proteins as antigens to develop robust immunogenicity. Construction of the vaccine (LSDV-SARS2-S,N) was confirmed by polymerase chain reaction (PCR) amplification and sequencing. In vitro characterization confirmed that cells infected with LSDV-SARS2-S,N expressed SARS-CoV-2 spike and nucleocapsid protein. In BALB/c mice, the vaccine elicited high magnitude IFN-γ ELISpot responses (spike: 2808 SFU/10^6^ splenocytes) and neutralizing antibodies (ID_50_ = 6552). Testing in hamsters, which emulate human COVID-19 disease progression, showed the development of high titers of neutralizing antibodies against the Wuhan and Delta SARS-CoV-2 variants (Wuhan ID_50_ = 2905; Delta ID_50_ = 4648). Additionally, hamsters vaccinated with LSDV-SARS2-S,N displayed significantly less weight loss, lung damage, and reduced viral RNA copies following SARS-CoV-2 infection with the Delta variant as compared to controls, demonstrating protection against disease. These data demonstrate that LSDV-vectored vaccines display promise as an effective SARS-CoV-2 vaccine and as a potential vaccine platform for communicable diseases in humans and animals. Further efficacy testing and immune response analysis, particularly in non-human primates, are warranted.

## 1. Introduction

Lumpy skin disease virus (LSDV) is a capripoxvirus that historically caused lumpy skin disease. It was restricted to Africa but has more recently caused outbreaks in Europe, the Middle East, and Asia. The virus is of economic importance as it causes serious disease in cattle and buffalo. Attenuated live vaccines are available to control outbreaks [1]. Replication of LSDV is limited to ruminants, and the restricted host range means it does not complete the replication cycle in primates, mice, rabbits, or hamsters [2,3]. It does not cause disease in immunocompromised mice and so is regarded as a safe vaccine vector [4]. Different poxviruses induce qualitatively and quantitatively distinct host immune responses, justifying the exploration of diverse poxvirus vectors. In an experiment examining the immune responses in mice 24 h after vaccination, LSDV induced greater interferon responses than modified vaccinia Ankara (MVA) and Avipoxviruses, as well as the greatest number of upregulated genes [5]. LSDV expressing an HIV polyprotein was immunogenic in Rhesus macaques at a 1000-fold lower dose than that of MVA. Both CD4+ and CD8+ T cell responses were induced, rather than a predominance of CD4+ T cells observed typically for poxvirus vectors [3,4]. Our group recently developed an improved version of the Neethling vaccine strain of LSDV designated nLSDVSODis-UCT [6], which has shown promising results as a vaccine vector for HIV, Bovine Ephemeral Fever, and East Coast Fever [7,8,9]. These studies have shown LSDV to be a promising candidate for a live host–range-restricted vaccine vector for human and animal diseases.

Most SARS-CoV-2 vaccine candidates contain the spike (S) protein or regions of the S protein, as it is considered the primary target of protective immunity. The S protein mediates SARS-CoV-2 entry into a host cell through binding to angiotensin-converting enzyme 2 (ACE2) and is the major target of neutralizing antibodies (nAbs), which have been shown to correlate with protection against SARS-CoV-2 [10]. One significant issue with S-based vaccines is the emergence of new variants of SARS-CoV-2, specifically Omicron and its subvariants, which exhibit notable differences in their S protein sequences compared to earlier strains targeted by the vaccines. The alterations in the S protein of these new variants have an impact on both neutralizing antibody and T cell responses [11,12]. Consequently, it has become evident that the existing COVID-19 vaccines may offer reduced protection against Omicron infection [13,14,15,16].

Patients without detectable levels of nAbs have recovered from SARS-CoV-2 infection, suggesting that cellular immunity also provides protection against SARS-CoV-2 [17,18,19]. Adaptive T-cell immunity has been demonstrated to be a crucial determinant of clinical outcomes and disease severity. T-cell memory typically involves the identification of viral proteins via the recognition of approximately 30 epitopes per person in a manner that is robust and well maintained over time [20]. This is in contrast to antibody-based B-cell immunity, which wanes over time in a manner that is well modeled and predictable [21]. Strong T-cell immunity can also help reduce the effects of escape mutations that allow new variants to escape antibody recognition, safeguarding against severe illness and lowering the burden of disease from increasingly diverse variants such as Omicron [16,20]. The present COVID-19 vaccines have been shown to provoke strong T-cell immunity, which is thought to have played a significant role in providing exceptional protection against hospitalization or fatality [22]. New and mixed vaccine regimens have the potential to further boost cellular and antibody-based responses and generally improve clinical outcomes and lower the burden of disease [16,23].

One strategy to improve long-term vaccine efficacy early on in a pandemic is to incorporate T-cell-targeted antigens from conserved regions of the viral genome, such as the SARS-CoV-2 nucleocapsid protein, which is known to have a lower mutation rate over time and is highly conserved among beta coronaviruses [24]. The nucleocapsid protein sequences of SARS-CoV (2003 epidemic) and SARS-CoV-2 (2020) show 90% conservation [25,26]. In contrast, the amino acid conservation of spike protein sequences was 76% [26]. Indeed, a particular immunodominant region from amino acid positions 102–110 within the SARS-CoV-2 nucleocapsid gene exhibits high potential for T-cell cross-reactivity with multiple human alpha and beta coronaviruses, giving some hope of protection against severe disease from a range of different coronaviruses [24]. Nucleocapsid is the most abundantly expressed SARS-CoV-2 protein and has been reported to be expressed on the surface of the virus, making it a potential target for antibody-dependent cellular cytotoxicity [27]. Vaccines expressing nucleocapsid induce robust antibody and T-cell responses and protect against severe disease in hamsters [28,29,30,31].

Exploiting the role of T cells in mediating disease control and infection is crucial for effective vaccine design, as it can increase the duration and breadth of vaccine effectiveness and improve clinical outcomes. Antibody-mediated immunity alone may negatively impact clinical outcome, as observed in the 2003 SARS-CoV outbreak, where high antibody levels were associated with increased inflammation [32].

In Southern Africa, the ability to develop effective vaccines locally for potential future outbreaks has become a high socio-economic priority [33]. In line with this overall idea, our group at the University of Cape Town developed a novel vaccine expressing both the SARS-CoV-2 spike and nucleocapsid proteins using our proprietary live attenuated nLSDVSODis-UCT vaccine vector. This vaccine could be used for both humans and animals.

## 2. Materials and Methods

### 2.1. Antibodies, Cell Lines, Viruses, Media, and Reagents

Rabbit polyclonal anti-spike (AB275759) and anti-nucleocapsid (AB273167) from Abcam (Cambridge, United Kingdom) were used as primary antibodies. Secondary antibody goat anti-rabbit-IgG (A3687, Sigma, Burlington, MA, USA) was used for Western blotting. Polyclonal goat anti-mouse IgG HRP (AB97023, Abcam, Cambridge, United Kingdom) was used to detect bound murine IgG in ELISAs.

Madin Darby bovine epithelial kidney cells (MDBK) (CCL-22™ ATCC^®^, USA), baby hamster kidney fibroblast 21 cells (BHK-21) (CCL-10™ ATCC, USA), and primary fetal lamb testes (LT) cells were cultured in Dulbecco’s modified Eagle’s medium with GlutaMAX™ (DMEM) (Thermo Fisher Scientific, USA) containing 10% fetal bovine serum (FBS) (Thermo Fisher Scientific, USA, or HyClone™ Cytiva, USA for LT cells) and 1X penicillin/streptomycin (1000 U/mL each, Lonza, Belgium), at 37 °C with 5% CO_2_.

nLSDVSODis-UCT was used as the LSDV backbone and for experimental controls. The virus is based on the Neethling vaccine strain originally obtained from Onderstepoort Biological Products (OBP, RSA), which was modified to encode a synthetic, full-length stabilized superoxide dismutase gene (SODis) [6]. The parent virus for our research, LSDV(SODis)BEFV-Gb, encodes the bovine ephemeral fever virus (BEFV) glycoprotein Gb antigen and eGFP, which are regulated by the respective vaccinia virus (VACV) mH5 and synthetic pSS poxvirus promoters, all inserted between ORFs 49 and 50 [8]. LSDV(SODis)BEFV-Gb was constructed using nLSDVSODis-UCT as the vector backbone. The BEFV Gb and eGFP genes were substituted with the SARS-CoV2, spike, nucleocapsid, and mCherry genes under the mH5, pLEO, and pSS promoters, respectively, to create a new recombinant LSDV-SARS2-S,N.

### 2.2. Transfer Vector Design

**Figure 1:**
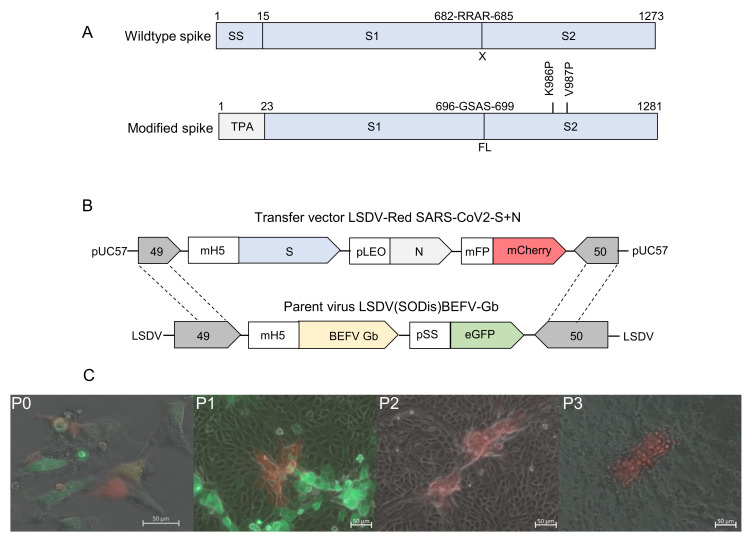
Construction of recombinant LSDV-SARS2-S,N. (**A**) Schematic diagrams of the wild type and modified SARS-CoV-2 spike antigens with amino acid residue positions labeled above. The native signal sequence (SS) was replaced with the tissue plasminogen activator signal sequence (TPA), the furin cleavage site (RRAR) was replaced with a short linker sequence (GSAS), and two stabilizing proline mutations were included (K986P and V987P). (**B**) Schematic diagram of the transfer vector LSDV-Red SARS-CoV2-S+N used for homologous recombination with the parent virus LSDV(SODis)BEFV-Gb genome between ORFs 49 and 50 to generate LSDV-SARS2-S,N. (**C**) Generation and passage of LSDV-SARS2-S,N. The recombinant virus (mCherry; red) was constructed in LT cells at passage 0 (P0) by infection with LSDV(SODis)BEFV-Gb (eGFP; green) and transfection with the transfer vector. P1 = first passage in MDBK cells, P2 = second passage in MDBK cells, P3 = third passage in MDBK cells. All scale bars = 50 µm.

The SARS-CoV-2 spike was designed based on the first publicly available genetic sequence (GenBank accession: MN908947.3), with the following modifications: the native leader sequence was replaced with the tissue plasminogen activator leader (GenBank: CAX11668.1) to aid entry to the secretory pathway; the furin cleavage site, RRAR between subunit 1 (S1) and subunit 2 (S2), was replaced with a linker sequence GSAS and two proline mutations (K986P and V987P) were introduced in S2 to stabilize the protein in the prefusion conformation (Figure 1A) [34]. No modifications were made to the nucleocapsid gene (GenBank accession: MN908947.3). Both genes were codon optimized for expression in humans and synthesized by GenScript (Hong Kong). The amino acid sequences of the genes described were retained; however, nucleotide sequences were modified to remove runs of four or more Cs and Gs, poxvirus transcription termination sites (T5NT), and unwanted restriction enzyme sites.

The transfer vector LSDV-Red SARS-CoV2-S+N (Figure 1B) was constructed to have the SARS-CoV-2 spike and nucleocapsid genes and the mCherry fluorescent marker gene, under the control of the respective VACV mH5, synthetic pLEO, and modified fowlpox promoters. The poxvirus transcription termination sequence was also included directly after the stop codon of each gene.

### 2.3. Construction of LSDV-SARS2-S,N

To make a recombinant LSDV expressing the SARS-CoV-2 spike and nucleocapsid proteins (LSDV-SARS2-S,N), primary fetal lamb testes (LT) cells were seeded at ~75% confluency in a 12-well plate (~4.3 × 10^5^ cells/well) and simultaneously transfected with 5.6 µL X-tremeGENE HP (Roche, Basel, Switzerland) plus 5.6 µg of transfer vector (LSDV-Red SARS-CoV2-S+N), linearized with XhoI and infected with recombinant LSDV(SODis)BEFV-Gb (MOI 0.08). The cells were then incubated for 3 days to allow for homologous recombination between the transfer vector and the parent virus (Figure 1B). The plate was then freeze-thawed twice, and 20 μL of the lysate was used to infect all the wells in eight 6-well plates of MDBK cells seeded with 3 × 10^5^ cells per well. Plates were incubated for 4 days, and red fluorescent foci marked for harvesting. The following day 18 foci were picked and frozen in 100 μL DMEM for further screening. One focus was freeze-thawed twice, and serial dilutions (2 × 10^−2^ to 1 × 10^−4^) of the lysate prepared for single-focus isolation in a 96-well plate. Each well was seeded with 4 x 10^5^ MDBK cells, and 10 μL of diluted virus lysate inoculated into the wells with 10 replicates of each dilution. The 96-well plate was incubated for 5 days, with daily inspection, to allow excess time for virus outgrowth and identification of any possible residual parent virus contamination. After 5 days, a single focus was identified and selected for viral expansion and screening. The plate was freeze-thawed twice, and the media from that well used to infect 2 wells in a 6-well plate, using 70 μL of lysate per well. DNA was extracted from one of these wells (Qiagen DNEasy Blood and TC kit) for polymerase chain reaction (PCR) screening and DNA sequencing, while the other was used to infect two T175 flasks of MDBK cells at 50% confluency. These were incubated for 7 days, after which a seed stock of recombinant LSDV-SARS2-S,N was prepared and titrated. Seed stocks were titrated by infecting MDBK cells in 96-well plates with serial dilutions of the stock (10^−1^ to 10^−12^ in DMEM), and tissue culture infectious dose at 50% infection (TCID_50_) was determined using the method described by Reed and Muench (1938) [35].

### 2.4. Preparation of LSDV-SARS2-S,N Stocks

High titer stocks of LSDV-SARS2-S,N were prepared by infecting MDBK cells in 175 cm^3^ flasks (Corning Inc^®^, New York, NY, USA) at MOIs 0.0025 to 0.005. Once all cells were infected, and about 50% of cells had lifted, the flasks were frozen and thawed twice, and lysates were clarified by low-speed centrifugation at 320× *g* for 10 min. Supernatants were placed into SS34 tubes and underlaid with 1–1.5 mL of 36% (*w*/*v*) sucrose diluted in 1x PBS. Viruses were pelleted by centrifugation at 27,000–39,000× *g* for 1–2 hrs at 4 °C. The pellets were resuspended in 1X PBS and stored at −80 °C until needed.

### 2.5. PCR Confirmation of the LSDV Recombinant

The insertion of the foreign gene cassette between LSDV ORFs 49 and 50 was confirmed by PCR, followed by agarose gel electrophoresis and Sanger DNA sequencing of the amplicon. The primer sequences used were 5′-GAGTGAAGCCTGGAACAT-3′ (forward primer) and 5′-ACTCTATCGCATCTGGAAACT-3′ (reverse primer). These generated fragment sizes of 1329 bp for control virus nLSDVSODis-UCT and 7200 bp for LSDV-SARS2-S,N. Phusion High-Fidelity enzyme was used with HF Buffer (New England BioLabs, Ipswich, MA, USA). The following thermocycling parameters were used for all PCR reactions: initial denaturation at 98 °C for 5 min followed by 40 cycles of denaturation at 98 °C for 30 s, annealing at 56 °C for 30 s, extension at 72 °C for 6 min and final extension at 72 °C for 10 min. PCR products were separated on 0.8% agarose gels containing 0.5 μg/mL ethidium–bromide by electrophoresis in 1xTBE buffer.

### 2.6. Confirmation of Spike and Nucleocapsid Expression

The expression of spike and nucleocapsid was confirmed by SDS PAGE and Western blot analysis. BHK-21 cells were infected with virus at MOI 0.01 in a 6-well plate. After 5 days, media was removed, and cells were lysed with 200 µL Glo Lysis Buffer (Promega, Madison, WI, USA) according to the manufacturer’s protocol. The cell lysates were clarified by centrifugation at 500× *g* for 5 min, and the pellets were resuspended in 200 µL PBS.

Samples were mixed with Laemmli buffer, boiled for 10 min at 95 °C, 30 µL per lane was loaded, and samples were separated on a 10% denaturing SDS PAGE and transferred to a polyvinylidene difluoride (PVDF) membrane (Bio-Rad, Hercules, CA, USA). The membranes were probed with 0.8 µg/mL rabbit anti-spike (Abcam: AB275759) and 0.9 µg/mL rabbit anti-nucleocapsid (Abcam: AB273167), followed by secondary antibody goat anti-rabbit-IgG conjugated to alkaline phosphatase, at 1:10,000 and detected with 5-bromo-4-chloro-3-indolylphosphate (BCIP)/nitroblue tetrazolium (NBT) phosphatase substrate (KPL, Seracare Life Sciences, Gaithersburg, MD, USA).

### 2.7. Mouse Immunizations

Mouse immunizations were conducted at the University of Cape Town’s Animal Research Facility in the Faculty of Health Sciences. All procedures were carried out in accordance with established protocols that were ratified by the UCT animal ethics committee (AEC 020_024). Female BALB/c mice (9 weeks old), bred at the University of Cape Town’s Animal Research Facility, were communally housed in TYPE 2 long cages and acclimatized to their environment for 10 days prior to any experimental procedures. Groups of 5 mice were randomly distributed into experimental and placebo groups. Mice were immunized with 50 µL of 10^7^ pfu of LSDV-SARS2-S,N or PBS mixed 1:1 with Alhydrogel^®^ adjuvant by intramuscular injection into the tibialis muscle on days 0, 21, and 42. Blood was drawn from the tail artery on days 0, 14, 35, and 56. Mice were sacrificed on day 56 by exsanguination.

### 2.8. IFN-γ ELISpot

The frequency of antigen-specific T cells was determined at the experimental endpoint by IFN-γ ELISpot. Briefly, 96-well plates were coated with 100 μL per well of 5 μg/mL anti-mouse IFN-γ antibody (BD Pharmingen) and incubated overnight at 4 °C. The plates were then blocked for 2 h in R10 media (RPMI 1640, 1% Pen/Strep, 2 mM L-Glutamine, 100 μL 50 mM 2-Mercaptoethanol, and 10% CTL-Test™ medium (CTL Immunogen)) at room temperature. Freshly harvested splenocytes were isolated from each mouse by mashing the spleen through a metal sieve strainer, with a 5 mL syringe plunger, into RPMI media. The splenocytes were washed three times with RPMI media and then lysed for 1 min with the addition of 1 mL Red Blood Cell (RBC) lysis buffer (Sigma R7757). Splenocytes were counted, and 5 × 10^5^ cells were added to each well of the 96-well plate. This was followed by the addition of either 100 μL of concanavalin A (1 μg/mL), spike peptide pools (2 μg/mL; GenScript: RP30020), nucleocapsid peptide pool (2 μg/mL; GenScript: RP30013), irrelevant peptide (2 μg/mL), or RPMI media (blank) to the plate in triplicate. The plate was incubated overnight at 37 °C and then sequentially washed three times, each with 100 μL of H_2_O and PBST (PBS containing 0.1% Tween 20 (Sigma, St Louis). The biotinylated detection antibody (BD Pharmingen) was added at 2 μg/mL and incubated for 2 h in the dark. The plate was then washed three times with PBST, and 100 μL of Avidin-horseradish peroxidase solution (BD Pharmingen) was added to the plate for an hour. Finally, the plate was washed 3× each with 100 μL PBST and then PBS before detecting with Nova Red substrate solution (Vector Labs, Newark, CA USA). The reaction was terminated by rinsing with tap water. The plate was dried overnight, and the number of Spot-Forming Units (SFU) was determined using a CTL Immunospot ELISpot reader.

### 2.9. Detection of Binding Antibodies to Spike

Antibody-binding ELISAs were conducted using pooled serum samples for each group. ELISA plates were coated overnight with 10 ng/well recombinant *E. coli*-produced SARS-CoV-2 spike protein (Invitrogen, RP-87668). Mouse sera were used in the primary incubation in a serial dilution range starting at 1:10 in PBS. PBST was used instead of PBS for all subsequent steps. Polyclonal goat anti-mouse IgG HRP (1:10,000) (Abcam, 97,023) was used to detect bound murine IgG, which recognized the coating antigen. Antibody endpoint titers were calculated from four-parameter logistic curves of duplicate data points with the threshold set as twice the geometric mean of the ELISA signal over the whole, matching pre-bleed serial dilution range. Data were plotted as mean ± SEM for the whole group. Experimental time points in the control group, which failed to yield a quantifiable endpoint titer, were attributed an arbitrary value of 10 to enable the data to be plotted.

### 2.10. Isolation, Propagation, and Titration of SARS-CoV-2, Delta Variant

All work involving live SARS-CoV-2 was performed inside an accredited Biosafety Level 3 facility in accordance with the safety regulations regarding risk level 3 pathogens [36]. SARS-CoV-2-positive patient samples were obtained from the National Health Laboratory Service (NHLS), Tygerberg, Cape Town, South Africa, and the lineage was confirmed to be SARS-CoV-2 Delta at Stellenbosch University (SU) as part of the Network for Genomic Surveillance in South Africa (NGS-SA) initiative [37].

Vero E6 cells were maintained in Dulbecco’s modified Eagle medium (DMEM) containing sodium pyruvate and L-glutamine (PAN Biotech, Aidenbach, Germany) with 10% fetal bovine serum (Gibco, Miami, FL, USA) and 1% each of non-essential amino acids (Lonza, Basel, Switzerland), amphotericin B (Gibco, Miami, FL, USA) and penicillin/streptomycin (PAN Biotech, Aidenbach, Germany). Vero E6 cells were grown at 37 °C and 5% CO_2_ and were passaged every 3–4 days.

For virus isolation, Vero E6 cells were seeded at 3.5 × 10^5^ cells/mL in a 6-well plate 18–24 h before infection. After one wash with 1xPBS (PAN Biotech, Aidenbach, Germany), the cells were inoculated with patient sample diluted 1:5 in DMEM. The inoculum was removed after one-hour incubation at room temperature, and the cells were washed once with 1xPBS before the addition of post-infection media (DMEM, 2%FBS, 1% each of non-essential amino acids, amphotericin B, penicillin/streptomycin). The cells were incubated at 37 °C with 5% CO_2_ and monitored daily for 3–7 days or until >90% cytopathic effect (CPE) was observed. The cell culture supernatant was then harvested and used to infect freshly-seeded Vero E6 cells to produce a second passage stock of the virus and then a third passage stock. The third passage stock was sequence confirmed at SU using Oxford Nanopore Technology, as described previously [38], to ensure no mutations had been introduced during passaging of the virus. The viral RNA load was quantified using a quantitative real-time PCR assay specific for the E gene, as described by Corman et al. [39], and the infectious virus titer was determined using a standard plaque assay on Vero E6 cells.

### 2.11. Hamster Vaccination and Intranasal Infection

Immunization and challenge experiments were carried out using 6–9-week-old male or female Syrian Golden Hamsters (*Mesocricetus auratus*). Hamsters for breeding were obtained from Janvier Labs, France, and bred for these experiments at the University of Cape Town’s Animal Research Facility. All experimental procedures were conducted in the Research Animal Facility at the University of Cape Town, in accordance with AEC 021_005. Animal immunizations took place under BSL-2 conditions, whereas viral challenge experiments were confined to the BSL-3 laboratory using the IsoRAT900 Biocontainment system. Prior to challenge experiments, the Delta variant of SARS-CoV-2 was tested to determine the optimal inoculum for infection, as described previously [40].

The vaccine challenge study was conducted using 5 hamsters per experimental group. Animals were immunized intramuscularly with 10^7^ pfu of LSDV per hamster on days 0 and 28. The experimental control group was immunized with an equivalent volume of PBS. Blood was drawn on days 0, 14, and 46. Vaccinated animals were transferred to the IsoRAT900 biocontainment system in the BSL3 laboratory on day 46 and were intranasally infected with 10^4^ pfu of the Delta variant of SARS-CoV-2 on day 49. Oropharyngeal swabs were collected on days 46 (prior to infection), 52 (3 days post infection), and 55 (5 days post infection). The weight of the hamsters was recorded prior to both vaccination and infection, and then monitored daily following infection, until the experimental endpoint. The experiment was terminated on day 55. Lungs were collected in 10% buffered formalin.

### 2.12. Neutralization Assay

Neutralizing antibodies against wild type/Wuhan and Delta viruses were quantified at weeks 0, 14, and 46 using a pseudovirus neutralization assay, as described previously [41]. SARS-CoV-2 pseudovirions were generated in HEK-293T cells by co-transfection of plasmids pNL4-3.Luc.R-.E- (aidsreagent #3418) and pcDNA3.3-SARS-CoV-2-spike Δ18 (Wuhan strain) [33,42] or pcDNA3.3-SARS-CoV-2-spike Δ18—Delta (Delta variant) [40], respectively. Neutralization titers were reflected as the half-maximal inhibitory dilution for each plasma sample.

### 2.13. Determination of SARS-CoV-2 RNA Viral Loads

SARS-CoV-2 RNA viral loads were determined from nasal swabs sampled in 500 µL QVL Lysis Buffer containing 10 µg/mL carrier RNA (Poly A) (Omega Bio-Tek). MS2 Phage Control (TaqMan™ 2019-nCoV Control Kit v2 (Applied Biosystems)) was added to each sample prior to RNA extraction as an internal positive control. RNA isolation was performed using the E.Z.N.A.^®^ Viral RNA Kit (Omega Bio-Tek). This was followed by multiplex qRT-PCR of the isolated RNA on a QuantStudio™ 7 Flex Real-Time PCR System (Thermo Fisher) using the TaqMan™ 2019-nCoV Control Kit v2 (Applied Biosystems), including serial dilutions of known SARS-CoV-2 viral RNA ranging from 1 × 10^4^ copies/µL to 1 × 10^0^ copies/µL. Viral loads were calculated based on the standard curve and expressed as copies/µL.

### 2.14. Histopathology

The lungs were sectioned and stained routinely with H&E for histopathology. Tissue was evaluated by light microscopy for evidence of necrosis/inflammation and/or repair/fibrosis. Lesions in the lung were graded as either absent (0), minimal (1), mild (2), moderate (3), marked (4), or severe (5). The severity of histopathology was graded based on observations of (1) lympho-plasmacytic infiltration, (2) bronchiolitis/peribronchiolitis, alveolitis and bullous emphysema, (3) vasculitis/perivasculitis, and (4) atelectasis.

### 2.15. Statistical Analyses

All statistical analyses were conducted using GraphPad Prism 9. Statistical comparisons between groups over time were analyzed using a two-way ANOVA test, whereas statistical comparisons between two groups at a single time point were made using a multiple unpaired t-test. A *p*-value < 0.05 was considered the threshold of significance for all statistical tests. The half-maximal inhibitor dilution (ID_50_) of sera samples was determined using non-linear regression.

## 3. Results

### 3.1. Construction and Confirmation of LSDV-Vectored SARS-CoV-2 Vaccine

Spike and nucleocapsid amino acid sequences were derived from the SARS-CoV-2 Wuhan isolate (GenBank MN908947.3). The following modifications were made to the spike protein: the native leader sequence was replaced with the tissue plasminogen activator leader sequence to aid entry to the secretory pathway; the furin cleavage site was replaced with a short linker sequence, and two proline mutations (K986P & V987P) were introduced to stabilize the protein in the prefusion conformation (Figure 1A) [34]. No modifications were made to the nucleocapsid.

To construct LSDV-SARS2-S,N, a transfer vector was designed containing the modified spike gene under the control of the mH5 promoter, the nucleocapsid gene under the control of the pLEO promoter, and the mCherry marker gene under the control of the modified fowl poxvirus promoter (Figure 1B). To construct the virus, at passage 0 (P0), LT cells were infected with the parent virus LSDV(SODis)BEFV-Gb and transfected with the transfer vector to enable homologous recombination to occur (Figure 1B,C). LSDV-SARS2-S,N was isolated by repeated passage of red fluorescing foci in MDBK cells until no green fluorescing foci (parent virus) were seen.

**Figure 2:**
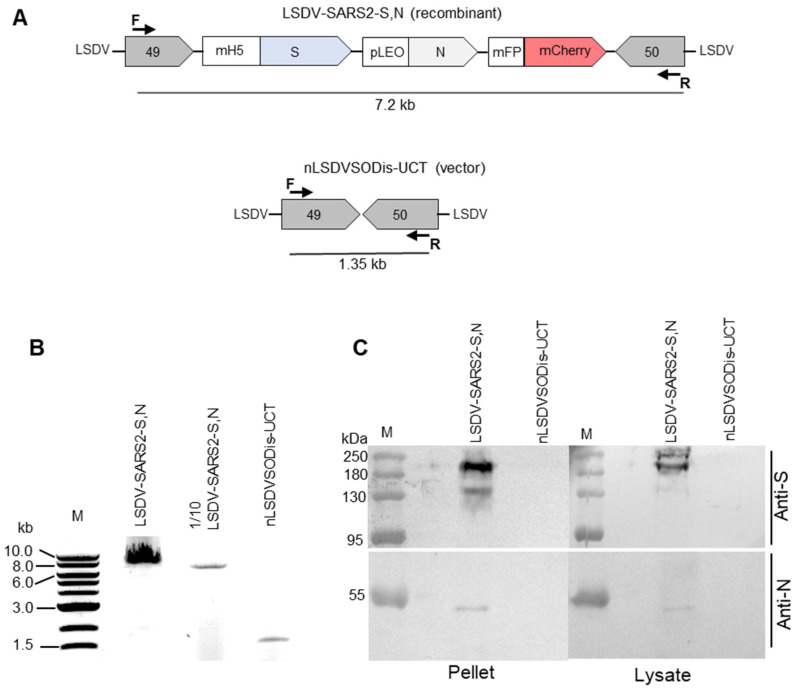
Characterization of LSDV-SARS2-S,N. (**A**) Schematic diagram showing the PCR product sizes expected to be amplified by the forward (F) and reverse (R) primers from LSDV-SARS2-S,N and nLSDVSODis-UCT. (**B**) Gel electrophoresis of PCR products from samples extracted from MDBK cells infected with LSDV-SARS2-S,N or nLSDVSODis-UCT. One-tenth of the sample from the first lane was loaded in the second lane. (**C**) SDS PAGE and Western blot of samples from infected MDBK cells. The membrane was cut in half and probed with either anti-spike (anti-S) or anti-nucleocapsid (anti-N) antibody.

The new recombinant LSDV-SARS2-S,N was confirmed to be correct by PCR (Figure 2A,B) and Sanger sequencing of the gene cassette inserted between LSDV ORFs 49 and 50. Expression of the spike and nucleocapsid proteins by cells infected with LSDV-SARS2-S,N was confirmed by SDS PAGE and Western blot analysis (Figure 2C). Two bands were detected corresponding to the full-length spike protein at just over and just under 180 kDa. This is probably due to differences in glycosylation states. A single band corresponding to the nucleocapsid protein at approximately 50 kDa was seen. Proteins of these sizes were not expressed by the negative control nLSDVSODis-UCT.

### 3.2. Evaluation of the Immunogenicity of LSDV-SARS2-S,N in BALB/c Mice

**Figure 3:**
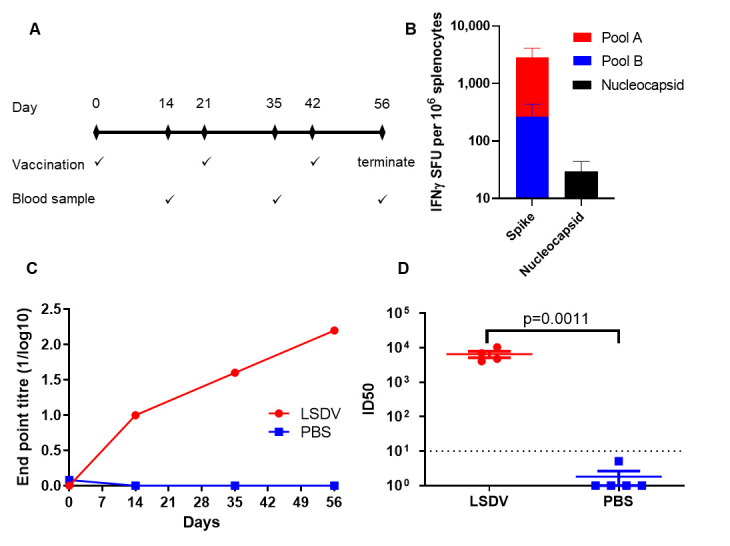
Immunogenicity of LSDV-SARS2-S,N in mice. (**A**) Schematic of the timing of immunizations and blood draws. (**B**) Frequency of antigen-specific T cells recognizing SARS-CoV-2 peptide pools. (SFU, spot-forming units). (**C**) Quantification of spike-specific antibodies by ELISA over the course of the experiment. The antibody titers are presented for pooled sera at each time point. (**D**) Terminal neutralizing antibody titers against the matched virus from which the vaccines were derived. (ID_50_, half maximal inhibitory dilution).

The immunogenicity of LSDV-SARS2-S,N was tested in mice (Figure 3A). Mice received three doses of 10^7^ pfu LSDV-SARS2-S,N every three weeks. Robust IFN-γ ELISpot responses were seen to the spike protein with a cumulative response of ~2808 SFU/10^6^ splenocytes (Figure 3B). Most of the response, ~2546 SFU/10^6^ splenocytes, was directed to peptide pool A, which largely consists of peptides from the S1 subunit of the spike protein. Only a small portion of the responses was peptide pool B (~262 SFU/10^6^ splenocytes), which consists mainly of peptides from the S2 subunit. IFN-γ ELISpot responses to the nucleocapsid were more modest at ~30 SFU/10^6^ splenocytes (Figure 3B).

Spike-binding antibodies were quantified with pooled sera to determine the endpoint titers after each immunization despite the low volume available for each bleed. Immunized mice developed robust binding antibodies that were detectable after the first immunization, which were boosted by approximately half a log after each subsequent inoculation (Figure 3C). Reassuringly, a negligible signal was observed in the control group comprising mice that were immunized with PBS.

All the mice in the LSDV-SARS2-S,N-vaccinated group developed high titers of neutralizing antibodies (6552 ID_50_) at the final bleed against the autologous Wuhan pseudovirion, with a negligible response observed from the PBS control (Figure 3D).

### 3.3. Immunogenicity of LSDV-SARS2-S,N in Hamsters and Protection from SARS-CoV-2 Challenge

**Figure 4:**
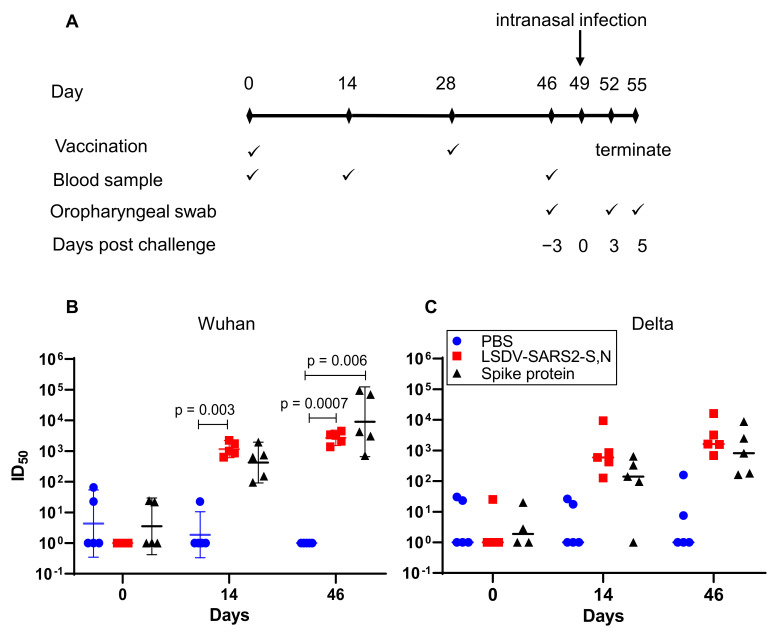
Immunogenicity of LSDV-SARS2-S,N in hamsters. (**A**) Immunization schematic depicting the timing of immunizations and sampling during the experiment. (**B**) Neutralizing antibody titers (ID_50_ = half maximal inhibitory dilution) against the matched wildtype (Wuhan) viral isolate. (**C**) Neutralizing antibody titers (ID_50_) against heterologous Delta virus. Pre-bleed = day 0, first bleed = day 14, final bleed = day 46. Geometric means of neutralizing antibody responses with 95% confidence intervals are shown.

Hamsters display clinical signs and symptoms similar to those seen in humans, such as weight loss, respiratory distress, lung damage, and viral shedding from the respiratory tract when infected with SARS-CoV-2 [43]. In contrast, mice generally show milder or no apparent clinical signs upon SARS-CoV-2 infection. Thus, hamsters are the preferred small animal model for SARS-CoV-2 infection and disease. Hamsters were immunized with two doses of LSDV-SARS2-S,N (10^7^ pfu) or PBS, four weeks apart (Figure 4A). Results from a previous publication where hamsters were vaccinated with 5 μg of spike protein, formulated 1:1 in Alhydrogel^®^ adjuvant were included as a positive control [40]. These experiments were all carried out at the same time. All the hamsters vaccinated with LSDV-SARS2-S,N developed neutralizing antibodies against the matched Wuhan virus (ID_50_ = 1292) and the heterologous Delta virus (ID_50_ = 2274) after a single inoculation and were boosted by the second vaccination (Figure 4B,C, Wuhan ID_50_ = 2905; Delta ID_50_ = 4648). The neutralizing antibody titers elicited by LSDV-SARS2-S,N were similar to those elicited by the spike protein (Figure 4B,C). Interestingly, neutralizing antibody titers to the heterologous Delta virus were higher than those to the autologous Wuhan virus; however, this was influenced by a particularly strong titer from one hamster to Delta. It is further noted that the variance in these titers between animals was also greater.

**Figure 5:**
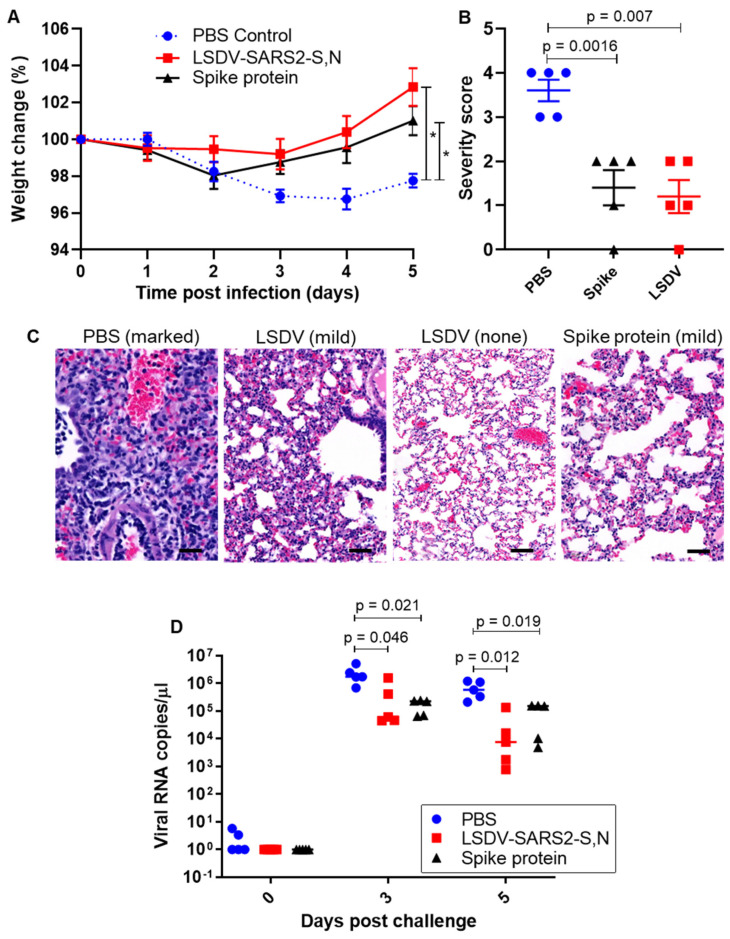
Vaccine-mediated protection against heterologous challenge. (**A**) Change in weight following experimental challenge with SARS-CoV-2 Delta (10^4^ pfu) 2-way ANOVA. * = *p* < 0.05 (**B**) Grading of lung pathology following challenge (t-test). (**C**) Representative images of histopathology findings in lung sections. Scale bars = 50 µm. (**D**) SARS-CoV-2 RNA viral load in the lungs, following challenge (t-test).

Three weeks after the second vaccination, the hamsters were challenged intranasally with Delta SARS-CoV-2 (10^4^ pfu) to evaluate the ability of the LSDV recombinant vaccine to protect from the heterologous viral challenge (Figure 4A). The Delta variant of SARS-CoV-2 was used, as this was the predominant variant circulating at the time of this experiment, and we wanted to determine whether vaccines based on the original wild-type variant provided protection against Delta. Hamsters vaccinated with LSDV-SARS2-S,N lost little or no weight after challenge and had gained an average of ~2.85% body weight by five days post challenge (Figure 5A). In contrast, hamsters from the PBS control group had lost ~3.25% body weight on average by day four post challenge and weighed ~2.25% less on average by the experimental endpoint (Figure 5A). Vaccination with LSDV-SARS2-S,N provided significant protection against weight loss, as compared to the mock vaccinated PBS control group (*p* < 0.0001). No significant differences in weight loss were seen between the LSDV-vaccinated group and the control group vaccinated with the spike protein. Weight loss in SARS-CoV-2-infected hamsters has been shown to be a good measure of disease [43].

To evaluate whether vaccination with LSDV-SARS2-S,N could prevent or minimize tissue damage from SARS-CoV-2 infection, histopathological analysis was carried out on the hamsters’ lungs five days post-SARS-CoV-2 infection. Pathology of lung tissue following challenge was graded based on the observed severity, which was reflected as a numerical score of 0–5, where 0 indicated that no significant histological changes were observed and 5 indicated severe changes (Figure 5B). Moderate to marked microscopic lesions were observed in all unvaccinated animals. In contrast, one of the hamsters vaccinated with LSDV-SARS2-S,N showed complete protection from challenge with no significant histological changes, and the remaining four only showed minimal changes (Figure 5B,C), resulting in a significant difference between the vaccinated group as compared to the PBS control. LSDV-SARS2-S,N vaccinated hamsters showed very similar lung pathology to that seen in hamsters vaccinated with the spike protein.

Similarly, viral RNA copies in oropharyngeal swabs were consistently lower in the vaccinated group as compared to the PBS control group (Figure 5D). At 5 days post infection, viral loads of the LSDV-vaccinated hamsters had decreased by approximately two orders of magnitude compared to the control group indicating a statistically significant (*p* = 0.012) improvement in viral control for the vaccinated hamsters (Figure 5D). Viral RNA copies in swabs of hamsters vaccinated with the spike protein were similar to the LSDV-vaccinated hamsters.

## 4. Discussion

Due to the ever-increasing demand for vaccines, the development of new vaccine vectors is needed, as the repeated use of viral vectors can result in anti-vector immunity [44]. Live virus vectors encoding foreign antigens offer several advantages over other strategies for COVID-19 vaccine development. They have the ability to stimulate both humoral and cellular immune responses, which is advantageous compared to subunit protein-based vaccines that typically induce antibody responses only. Due to their inherent immunogenicity, live virus vectors often do not require additional adjuvants to enhance immune responses. Furthermore, the active infection caused by these vectors facilitates efficient cellular uptake, unlike DNA vaccines that rely on translocating to the host nucleus [45,46,47]. Poxviruses have many features that make them ideal vectors for a SARS-CoV-2 vaccine. They are relatively easy and cheap to produce, have the capacity for insertion of up to 25 kbp of foreign DNA, can be freeze dried and thus do not require cold chain storage, and elicit long-lasting immune responses. Our group previously showed that an LSDV-vectored vaccine against HIV-1 induced comparable humoral immune responses to an MVA-vectored vaccine expressing the same HIV-1 antigens [48]. In this study, we constructed an LSDV-vectored vaccine against SARS-CoV-2. In contrast to most other SARS-CoV-2 vaccine candidates, which only target the spike protein, the candidate recombinant LSDV vaccine was designed to express both the spike and nucleocapsid antigens of SARS-CoV-2, as it has been proposed that next-generation SARS-CoV-2 vaccines could be improved by the addition of the N protein [24,26].

The course of the COVID-19 pandemic to date has demonstrated that SARS-CoV-2 is able to acquire mutations in its spike gene that allow it to escape neutralizing antibody immunity and alter the viruses R0 infectivity rate in a concerning manner [16,49]. This has also posed challenges for COVID-19 vaccines, which principally target the spike protein, as some of these spike variants have reduced sensitivity to vaccine-induced neutralization [12,50,51]. Clinical studies have also shown that early COVID-19 vaccines that only target the spike antigen derived from the Wuhan-like virus have decreased efficacy against subsequent variants such as Delta [52] and Omicron [53] variants. This supports the rationale to include the nucleocapsid in a COVID-19 vaccine as it is highly conserved and abundantly expressed, making it an ideal immunogen for the induction of broad, robust T-cell responses.

While there is strong evidence that neutralizing antibodies against the spike protein provide protection against SARS-CoV-2 infection, many studies also indicate that cellular immune responses play an important role in the clearance of SARS-CoV-2 and the alleviation of COVID-19 disease severity [20,22,54,55,56,57]. New SARS-CoV-2 variants with multiple mutations in the spike protein that can escape antibody neutralization and reduce vaccine-induced protection from infection are increasing. However, T-cell responses generated by vaccination or prior infection have been shown to be highly cross reactive with Omicron and other SARS-CoV-2 variants of concern [55,58]. These responses target T-cell epitopes located across the entire spike protein, suggesting that the SARS-CoV-2 viral evasion from T cells may be limited [59]. Antibody-seronegative-exposed family members and convalescent individuals with a history of asymptomatic and mild COVID-19 have been shown to have SARS-CoV-2-specific T cell responses, indicating that robust T-cell immunity might also protect individuals from SARS-CoV-2 infection [60]. In this study, we have shown that an LSDV-vectored COVID-19 vaccine induced robust T-cell responses to the SARS-CoV-2 spike protein and more modest responses to the nucleocapsid in mice. Ways of improving T-cell responses to the nucleocapsid should be investigated. These could include the use of a heterologous prime-boost strategy, which has been shown to elicit higher magnitude immune responses than a homologous recombinant LSDV regimen [3,4].

LSDV-SARS2-S,N induced high titers of neutralizing antibodies against the autologous Wuhan and heterologous Delta variant of SARS-CoV-2 in Syrian hamsters. Neutralizing antibody levels are highly predictive of immune protection from symptomatic SARS-CoV-2 infection [10]. Protective efficacy in convalescent sera from seven phase 3 clinical trials equated to in vitro neutralization titers of between 1:10 and 1:30 in most of the assays reported (although up to 1:200 in one assay) [10]. A single inoculation of LSDV-SARS2-S,N induced titers of neutralizing antibodies ranging from 1:634 to 1:2232 to the Wuhan variant and titers ranging from 1:126 to 1:9367 to the Delta variant. These increased to 1:1368 to 1:4468 and 1:682 to 1:16149 to the Wuhan and Delta variants, respectively, after a second inoculation. This protection by nAbs was supported following challenge, as minimal-to-no weight loss or histological changes in the lungs were seen in the hamsters vaccinated with LSDV-SARS2-S,N, indicating that they were protected from disease. Moreover, SARS-CoV-2 RNA viral copies in oropharyngeal swabs from vaccinated hamsters were 5.5-fold lower than the control group three days post infection and 21-fold lower by five days post infection. The more rapid reduction in viral copies from days three to five post infection in the LSDV-SARS2-S,N vaccinated hamsters as compared to the PBS control group could be due to the virologic control mediated by CD8+ T cells [20,56,58].

Although LSDV has shown promise as a candidate vaccine vector for cattle diseases, we propose this poxvirus vector could be exploited in the development of both human and animal vaccines as a replication-deficient virus that can infect nonpermissive hosts and express foreign genes [48]. Both humoral and cytotoxic immune responses are elicited by candidate recombinant LSDV vaccines.

In conclusion, the LSDV-SARS2-S,N candidate vaccine induced high titer neutralizing antibody responses in mice and hamsters to SARS-CoV-2 and provided protection against disease in hamsters, supporting the use of LSDV as an excellent viral vector for vaccines. Inclusion of a control group of animals vaccinated with the LSDV vector backbone, rather than PBS, to assess whether the viral vector induced some form of non-specific protection against SARS-CoV-2 would also be of interest. Evaluation of the protective efficacy of LSDV-SARS2-S,N against more divergent variants such as Omicron and in non-human primates with a more in-depth characterization of the cellular immune responses to both the spike and nucleocapsid antigens are warranted. The duration of the protective immune responses should also be monitored in future studies.

## Data Availability

All data provided in the paper.
